# Impact of anti-rheumatic treatment on immunogenicity of pandemic H1N1 influenza vaccine in patients with arthritis

**DOI:** 10.1186/ar4427

**Published:** 2014-01-02

**Authors:** Meliha C Kapetanovic, Lars-Erik Kristensen, Tore Saxne, Teodora Aktas, Andreas Mörner, Pierre Geborek

**Affiliations:** 1 Department of Clinical Sciences, Section of Rheumatology Lund, Lund University and Skåne University Hospital, Lund, Sweden; 2 Vaccinology Unit, Department of Diagnostics and Vaccinology, Swedish Institute for Communicable Disease Control, Solna, Sweden

## Abstract

**Introduction:**

An adjuvanted pandemic H1N1 influenza (pH1N1) vaccine (Pandemrix®) was reported as highly immunogenic resulting in seroconversion in 77 to 94% of adults after administration of a single dose. The aim of the study was to investigate the impact of different anti-rheumatic treatments on antibody response to pH1N1 vaccination in patients with rheumatoid arthritis (RA) and spondylarthropathy (SpA).

**Methods:**

Patients with arthritis (n = 291; mean age 57 years, 64% women) participated. Hemagglutination inhibition (HI) assay was performed on blood samples drawn before and after a mean (SD) of 8.3 (4) months following vaccination. A positive immune response i.e. seroconversion was defined as negative prevaccination serum and postvaccination HI titer ≥40 or a ≥4-fold increase in HI titer. All patients were divided into predefined groups based on diagnosis (RA or SpA) and ongoing treatment: methotrexate (MTX), anti-tumor necrosis factor (anti-TNF) as monotherapy, MTX combined with anti-TNF, other biologics (abatacept, rituximab, tocilizumab) and non-steroidal anti-inflammatory drugs (NSAIDs)/analgesics. Predictors of positive immune response were studied using logistic regression analysis.

**Results:**

The percentage of patients with positive immune response in the different treatment groups was: 1. RA on MTX 42%; 2. RA on anti-TNF monotherapy 53%; 3. RA on anti-TNF + MTX 43%; 4. RA on other biologics (abatacept 20%, rituximab 10% and tocilizumab 50%); 5. SpA on anti-TNF monotherapy 76%; 6. SpA on anti-TNF + MTX 47%; and 7. SpA on NSAIDs/analgesics 59%. RA patients on rituximab had significantly lower (P < 0.001) and SpA on anti-TNF monotherapy significantly better response rates compared to other treatment groups (*P* 0.001 to 0.033). Higher age (*P* < 0.001) predicted impaired immune response. Antibody titers 3 to 6 months after vaccination was generally lower compared to those within the first 3 months but no further decrease in titers were observed 6 to 22 months after vaccination.

**Conclusions:**

Rituximab treatment severely reduced antibody response to pH1N1 influenza vaccine. The other treatment groups showed acceptable antibody responses. Protective antibody titers could be detected up to 22 months after vaccination in the current patient population, with the exception of rituximab treated patients.

## Introduction

During the 2009 influenza pandemic a mass vaccination was performed across Europe. In Sweden free vaccination against pandemic H1N1 (pH1N1) influenza virus was offered to all residents. According to a report from the Swedish WHO National Influenza Centre published by The Swedish Institute for Communicable Disease Control, the estimated coverage of H1N1 vaccination in the entire country was 60%; ranging from 54% to 70% in different counties [1]. An inactivated, monovalent, split vaccine (Pandemrix®) containing 3.75 μg hemagglutinin (HA) and a squalene-based oil-in-water adjuvant system (AS03) was used [2]. The vaccine was reported as highly immunogenic resulting in seroconversion in 77% to 94% of adults after administration of a single dose [2-5]. Usage of the adjuvant was shown to improve antibody response of the inactivated vaccine but was associated with more local adverse effects [3,5,6] compared to unadjuvanted vaccine. Furthermore, persistence of immune response up to at least six months following a single dose of adjuvant vaccine was reported in adult healthy subjects including those 65-years-old and older [6-8]. In Sweden, all subjects receiving immunosuppressive drugs including biological remedies were considered to be at increased risk of complications from influenza infection and, therefore, immunization with two doses of the vaccine preferably administered 21 days apart was recommended by the Swedish National Board of Health and Welfare [9]. A number of studies have investigated the influence of different treatments on the immunogenicity of seasonal influenza and adjuvanted pH1N1 vaccines in patients with inflammatory rheumatic diseases with somewhat conflicting results [10-20]. Our group previously reported antibody response after seasonal influenza vaccine in rheumatoid arthritis patients (RA) treated with methotrexate (MTX) or anti-TNF remedies being as good as that of healthy controls [21].

We initially conducted an investigator-driven formal vaccine study with conjugated pneumococcal vaccine (Prevenar7) in rheumatoid arthritis (RA) and spondylarthropathy (SpA) patients including long term antibody development [22,23]. The mass vaccination against pH1N1 largely coincided with the Prevenar7 study. Therefore, the aim of the present study was to investigate immune responses against an adjuvanted pH1N1 vaccine in relation to timing (1 to 22 months after vaccination) and dosing (single/two doses) as well as predictors of positive immune response in defined cohorts of RA and SpA patients on different treatments including biologic remedies. We also aimed to assess tolerability of the pH1N1 vaccine in patients with RA and SpA treated in clinical practice.

## Methods

**Figure 1:**
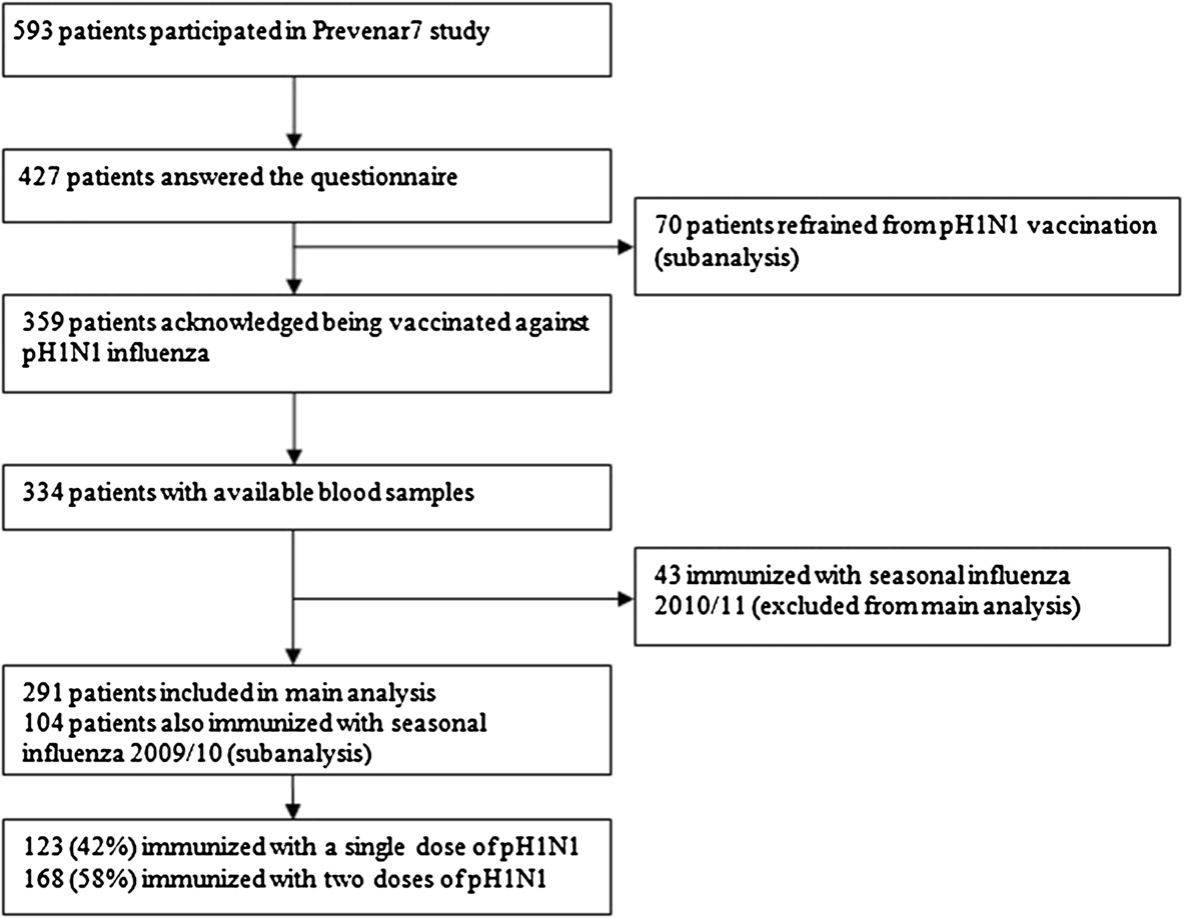
Flowchart of the study population.

Consecutive arthritis patients monitored at out-patient rheumatology units of the Department of Rheumatology, Skåne University Hospital, Lund and Malmö, Sweden who were participating in the Prevenar7 vaccination study [22] were asked to answer a questionnaire regarding pH1N1 influenza vaccination status, including the number of doses received and tolerability of the vaccine. Information on seasonal influenza vaccination status in 2009/2010 and, when appropriate, the winter season 2010/2011 (when pH1N1 was included in the seasonal vaccine) was also collected. A flowchart of the study population is shown in Figure 1.

All patients were immunized with a single dose or two doses of monovalent, split, adjuvanted, pandemic influenza A/H1N1 vaccine (Pandemrix®) containing 3.75 μg HA and AS03 [2]. Vaccination was performed during the winter season 2009/2010. In spite of recommendations some immunosuppressed patients refrained from the second dose of vaccine. When two doses were given, the immunization was performed at least 21 days apart.

Antibody response was determined using hemagglutination inhibition (HI) assay [24] on stored blood samples drawn prior to autumn 2009 and after mean (SD) 8.3 (5) months following vaccination. All sera were titrated simultaneously and blinded for patients, treatment characteristics and vaccination status.

The seroprotection rate, that is, a percentage of patients with postvaccination titers ≥40 was calculated. Seroconversion was defined as negative prevaccination serum and postvaccination HI titer ≥40 or a ≥4-fold increase in HI titer. Pre- and postvaccination geometric mean titers and geometric mean fold increase were calculated. The percentages of patients who met EU Committee of Human Medicinal Products (CHMP) licensing criteria for assessment of influenza vaccines were calculated. According to these criteria sufficient protection against infection in healthy adults 18 to 60-years old (>60-years-old) is assumed if at least one of three criteria is fulfilled: seroprotection rate 70% (60%); seroconversion rate 40% (30%) or mean increase in geometric mean titer (geometric mean fold increase) >2.5 (3) [25].

The study was approved by the Regional Ethics Review Board at Lund University (file numbers 97/2007 and 519/2009). Informed written consent was obtained from all subjects before study entry.

### Statistical analysis

Non-parametric tests were generally used. Differences in positive immune response between treatment groups were calculated using the Chi2 test. Geometric mean antibody titers (GMT) were calculated using log transformed antibody levels. Possible predictors of positive immune response were studied using binary logistic regression analysis.

## Results

### All patients

Patient selection is outlined in the flow chart in Figure 1. The Prevenar7 vaccine study cohort consists of 505 patients with RA or SpA participating in the original study and 88 additional subsequently included patients treated with other biologics than anti-TNF remedies. The inclusion criteria for that study were that anti-rheumatic treatment had not been changed for at least four weeks before the inclusion [22]. Biological remedies were administered according to daily clinical practice and at least two treatment courses had been given before the study entry. Treatment with at least one anti-TNF drug before switching to other biological modalities (abatacept, rituximab or tocilizumab) was mandatory at our Department when the study was initiated.

Of all 593 patients, 427 answered the questionnaire regarding pH1N1 influenza vaccine. Seventy had refrained from pH1N1 vaccination while 359 patients acknowledged being pH1N1 vaccinated with at least one dose of inactivated adjuvant vaccine. Of these, there were 334 with stored serum samples before and after pH1N1 vaccination. Since seasonal influenza vaccine for 2010/2011 contained a pH1N1 virus strain, patients immunized with that vaccine were excluded from the analysis if their blood samples were collected after that vaccination. In total, 291 patients were included in the main analysis. Based on diagnosis and ongoing anti-rheumatic treatments patients were stratified into the following groups: 1) RA on MTX (n = 50); 2) RA on anti-TNF as monotherapy (n = 38); 3) RA on anti-TNF + MTX (n = 53); 4) RA on other biologics (abatacept (n = 5), rituximab (n = 10), tocilizumab (n = 2)); 5) SpA on anti-TNF as monotherapy (n = 41); 6) SpA on anti-TNF + MTX (n = 51); and 7) SpA on NSAIDs/analgesics (controls, n = 41). Patient characteristics for different treatment groups are given in Table 1.

**Table 1:** Demographic and disease characteristics of the study population

	**All patients (number = 291)**	**RA on MTX (number = 50)**	**RA on anti-TNF monotherapy (number = 38)**	**RA on anti-TNF + MTX (number = 53)**	**RA on abatacept (number = 5)**	**RA on rituximab (number = 10)**	**RA on tocilizumab (number = 2)**	**SpA on anti-TNF monotherapy (number = 41)**	**SpA on anti-TNF + MTX (number = 51)**	**SpA on NSAIDs/ analgesics controls (number = 41)**
Age (years) mean (range)	57 (23 to 87)	62.3 (26 to 87)	62.4 (32 to 86)	61.1 (41 to 83)	55.2 (43 to 66)	62.3 (43 to 79)	63.5 (55 to 72)	49.0 (2 to 69)	52.6 (26 to 71)	52.2 (23 to 72)
Gender (% female)	64	78	84	80	86	80	100	37	51	51
Disease duration (years) mean (range)	16.0 (1 to 55)	12.5 (1 to 41)	21.1 (3 to 46)	18.2 (2 to 48)	12.4 (6 to 24)	22.3 (5 to 55)	21.5 (15 to 28)	16.6 (1 to 36)	13.1 (1 to 42)	14.3 (1 to 37)
RF positive (%)	43	76	87	76	100	80	100	-----	-----	----
Anti-CCP positive (%)	43	82	74	83	80	80	50	------	-----	……
HLA-B27 positve (%)	22.3	-----	------	-----	------	-------	------	56	28	68

MTX, methotrexate; NSAIDS, non-steroidal anti-inflammatory drugs; RA, rheumatoid arthritis; SpA, spondylarthropathy.

### Timing after pH1N1 vaccination

**Figure 2:**
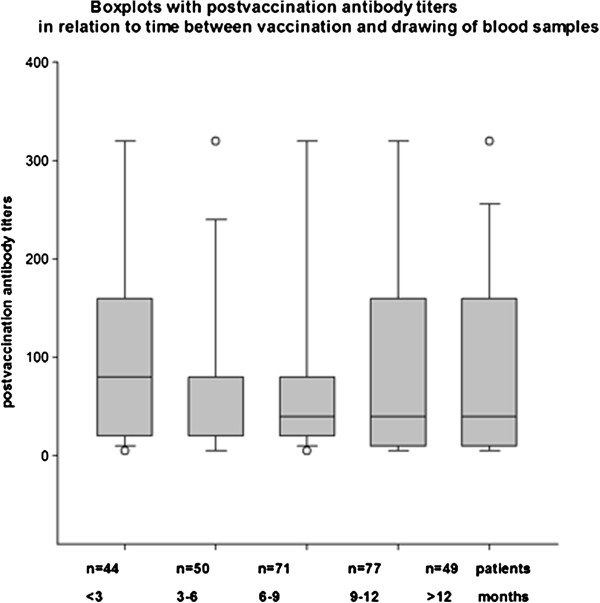
Box plots with postvaccination antibody titers in relation to the time between vaccination and drawing of blood samples.

Blood samples were drawn prior to and after mean 8.3 months (SD 4); range 1 to 22 months following vaccination. During this time period biological treatments were continued according to clinical practice and rituximab-treated patients may have received more than one treatment course. Figure 2 shows the box plots with postvaccination antibody titers in relation to time elapsed between vaccination and blood samplings. Three to six months after vaccination antibody titers were generally lower compared to those collected within the first three months but no further decrease in these titers was observed in patients whose samples were collected >6 months after the vaccination. The time period between vaccination and retrieval of blood samples did not influence positive antibody response (*P* = 0.306).

### Immune response after pH1N1 vaccination

A positive immune response, that is, seroconversion, was defined as prevaccination antibody titers <10 and postvaccination HI titer ≥40 or a ≥4-fold increase in HI titer.

**Figure 3:**
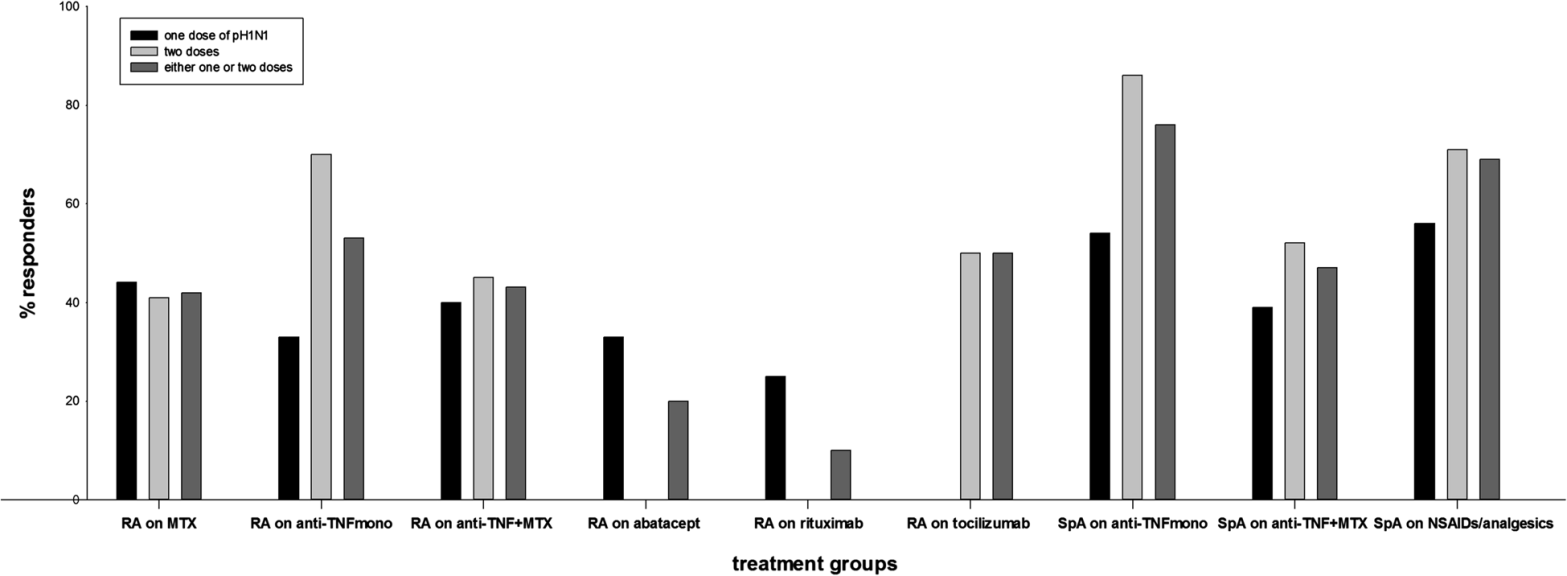
**Proportion (%) of patients with positive immune response, that is seroconversion (defined as prevaccination titers <10 and postvaccination HI titers ≥40 or a ≥4-fold increase in HI titers), in different treatment groups stratified according to the number of doses of vaccine.** HI, hemagglutination inhibition.

Table 2 summarizes prevaccination- and postvaccination GMT, geometric mean fold increase, number (%) of patients with prevaccination and postvaccination antibody titers ≥40 (seroprotection) and positive immune response (seroconversion) in the entire study population and different treatment groups after immunization with a single dose, two doses, or all immunized patients irrespective of doses of the pH1N1 vaccine. The percentage of patients immunized with seasonal influenza vaccine in 2009/2010 is also given. Figure 3 illustrates the proportion (%) of patients with a positive immune response in different treatment groups stratified according to the number of vaccine doses administered.

**Table 2:** Immune response following vaccination with single dose, two doses or irrespective of number of doses

	**All patients (number = 291)**	**RA on MTX (number = 50)**	**RA on anti-TNF monotherapy (number = 38)**	**RA on anti-TNF + MTX (number = 53)**	**RA on abatacept (number = 5)**	**RA on rituximab (number = 10)**	**RA on tocilizumab (number = 2)**	**SpA on anti-TNF monotherapy (number = 41)**	**SpA on anti-TNF + MTX (number = 51)**	**SpA on NSAIDs/ analgesics controls (number = 41)**
**Patients immunized with a single dose of the vaccine (number = 123)**										
Number of patients	123	18	18	15	3	4	0	13	18	34
GMT prevaccination mean (95% CI)	9.3 (7.2 to 12.1)	6.8 (5.1 to 9.1)	10.8 (6.4 to 18.3)	6.9 (5 to 9.5)	16 (1.1 to 220)	7.1 (2.3 to 21.3)	---	8.5 (5.4 to 13.5)	10 (6.9 to 14.5)	8.8 (6.7 to 11.7)
GMT postvaccination mean (95% CI)	52.4 (34.3 to 80.1)	34.3 (16.6 to 70.8)	30.5 (14.6 to 64)	19.1 (11.2 to 32.5)	49 (2 to 790)	14.1 (3.4 to 59)	---	40 (21.6 to 74)	23.3 (12.7 to 43)	46.1 (29 to 73.9)
Geometric mean fold increase (95% CI)	5.6 (3.6 to 8.8)	5 (2.1 to 12.2)	2.8 (1.6 to 5.1)	2.8 (1.4 to 5.4)	2.5 (0.3 to 18.4)	2 (0.4 to 9.5)	---	4.7 (2.1 to 10.5)	2.3 (1.1 to 4.8)	5.2 (3.2 to 8.4)
Number (% ) of patients with prevaccination titer ≥40	8 (7%)	0	2 (11%)	0	1 (33%)	0	---	1 (8%)	1 (6%)	3 (9%)
Number (% ) of patients with postvaccination titer ≥40 (seroprotection)	61 (49.1%)	9 (50%)	8 (44%)	6 (40%)	1 (33%)	1 (25%)	---	7 (54%)	8 (44%)	21 (62%)
Number (%) of patients with positive immune response (seroconversion)^a^	55 (45%)	8 (44%)	6 (33%)	6 (40%)	1 (33%)	1 (25%)	----	7 (54%)	7 (39%)	19 (56%)
% immunized with seasonal influenza vaccine (2009/2010)	22	33	44	20	0	0	----	0	11	24
^a^No significant differences between treatment groups.										
**Patients immunized with two doses of the vaccine (number = 168)**										
Number of patients	168	32	20	38	2	6	2	28	33	7
GMT prevaccination mean (95% CI)	9.2 (8.2 to 10.4)	9.8 (7.1 to 13.4)	8.7 (6.3 to 12.1)	6.8 (5.6 to 8.3)	20 (3.3 to 121)	7.9 (3.7 to 16.8)	28 (0.3 to 2312)	9.5 (7.1 to 12.7)	10.7 (8.1 to 14.1)	12.2 (5 to 29.5)
GMT postvaccination mean (95% CI)	50 (40.4 to 61.8)	40 (23.9 to 67.5)	72 (33 to 159)	34 (21.5 to 54)	33.6 (6.4 to 176)	8.9 (3.8 to 21)	113	102.5 (69 to 153)	48.3 (32 to 73.4)	97.5 (33 to 291)
Geometric mean fold increase (95% CI)	5.4 (4.3 to 6.8)	4.1 (2.5 to 6.7)	8.3 (3.7 to 18.5)	5 (3–8.3)	2	1.1 (0.5 to 2.6)	4 (0---)	11 (6.7 to 17.3)	4.5 (2.6 to 7.8)	8 (1.5 tp\o 43.6)
N (% ) of patients with prevaccination titer ≥40	17 (10%)	6 (19%)	1 (5%)	1 (3%)	1 (50%)	0	1 (50%)	2 (7%)	3 (9%)	2 (29%)
N (% ) of patients with postvaccination titer ≥40	106 (63%)	16 (50%)	14 (70%)	18 (48%)	1 (50%)	1 (17%)	2 (100%)	24 (86%)	24 (73%)	6 (86%)
N (%) of patients with positive immune response (seroconversion)	91 (54%)	13 (41%)^b^	14 (70%)	17 (45%)	0	0	1 (50%)	24 (86%)^a^	17 (52%)	5 (71%)
% immunized with seasonal influenza vaccine (2009/2010)	46%	59%	40%	51%	100	50	100	32	36	43
^a^*P* = 0.003 compared to RA on MTX; *P* = 0.033 compared to RA on anti-TNF monotherapy; *P* = 0.001 compared to RA on anti-TNF + MTX; *P* = 0.005 compared to SpA on antiTNF + MTX; ^b^*P* = 0.039 versus RA on anti-TNF monotherapy (Chi2 test).										
**All vaccinated patients regardless of number of doses (number = 291)**										
Number of patients	291	50	38	53	5	10	2	41	51	41
GMT prevaccination mean (95% CI)	9 (8.2 to 9.8)	8.6 (6.8 to 10.8)	9.6 (7.2 to 12.9)	6.8 (5.8 to 8)	17.4 (3.7 to 81.2)	7.6 (4.7 to 12.2)	28.3 -----	9.2 (7.3 to 11.6)	10.4 (8.4 to 12.9)	9.3 (7.2 to 12.1)
GMT postvaccination mean (95% CI)	41.4 (35.3 to 48.4)	37.8 (25.1 to 57)	48 (28.1 to 82.1)	28.8 (20.2 to 41.3)	40 (11.8 to 135)	10.7 (5.9 to 19.4)	113.1 -----	76 (53.5 to 105)	37.4 (26.4 to 53)	52.4 (34.3 to 80.1)
Geometric mean fold increase (95% CI)	4.6 (3.9 to 5-5)	4.4 (2.9 to 6.9)	5 (3 to 8.3)	4.2 (2.8 to 6.3)	2.3 (0.9 to 5.9)	1.4 (0.8 to 2.6)	4	8.3 (5.5 to 12.5)	3.6 (2.3 to 5.5)	5.6 (3.6 to 8.8)
N (% ) of patients with prevaccination titer ≥40	25 (9%)	6 (12%)	3 (8%)	1 (2%)	2 (40%)	0	1 (50%)	3 (7%)	4 (8%)	5 (12%)
N (% ) of patients with postvaccination titer ≥40 (seroprotection)	167 (57%)	25 (50%)	22 (58%)	24 (45%)	2 (40%)	2 (20%)	2 (100%)	31 (76%)	33 (63%)	27 (66%)
N (%) of patients with positive immune response (seroconversion)	146 (50%)	21 (42%)^b^	20 (53%)^c^	23 (43%)^d^	1 (20%)	1 (10%)	1 (50%)	31 (76%)^a^	24 (47%)	24 (59%)
% immunized with seasonal influenza vaccine (2009/2010)	104 (36%)	50%	42%	42%	40%	30%	100%	22%	28%	27%
^a^*P* = 0.001 compared to RA on MTX; *P* = 0.033 compared to RA on anti-TNF as monotherapy; *P* = 0.002 compared to RA on anti-TNF + MTX; *P* = 0.011 compared to RA on abatacept; *P* < 0.001 compared to RA on rituximab; *P* = 0.006 compared to SpA on anti-TNF + MTX; ^b^*P* = 0.05 compared to RA on rituximab; ^c^*P* = 0.013 compared to RA on rituximab; *P* = 0.033 compared to SpA on ^d^*P* = 0.046 compared to RA on rituximab (Chi2 test/Fisher’s exact test).										

Positive immune response, that is seroconversion, was defined as prevaccination titers <10 and postvaccination HI titers ≥40 or a ≥4-fold increase in HI titers. Percentages of patients vaccinated against seasonal influenza during the 2009/2010 winter season are also given. CI, confidence interval; GMT, geometricial mean antibody titer; HI, hemagglutination inhibition; MTX, methotrexate; NSAIDs, non-steroidal anti-inflammatory drugs; RA, rheumatoid arthritis; SpA, spondylarthropathy.

### Immunization with a single dose or two doses of the vaccine

Of the 291 patients, 123 (42%) received one dose of the vaccine. Patients treated with rituximab had significantly lower postvaccination GMT, lower mean increase in geometric mean titer compared to other treatment groups and only one patient (25%) with a positive immune response (seroconversion). Vaccination with a single dose did not meet any CHMP criteria for protection against infection in rituximab treated patients as a group. Otherwise, the proportion of patients with a positive immune response after receiving a single dose of vaccine was similar in all treatment groups.

Of the 291 patients, 168 (58%) received two doses of the vaccine. Compared to a single dose, immunization with two doses of the vaccine overall resulted in more patients with a positive immune response in all treatment groups except for RA patients on MTX and RA patients on rituximab. The proportion of MTX-treated RA patients with positive immune response after a single dose of the vaccine and after two doses were 45% and 41%, respectively. None of six RA patients on rituximab immunized with two doses had a positive immune response.

### All immunized patients (single and two doses together)

The percentage of patients with a positive immune response in the different treatment groups was: 1) RA on MTX, 42%; 2) RA on anti-TNF monotherapy, 53%; 3) RA on anti-TNF + MTX, 43%; 4) RA on other biologics (abatacept 20%, rituximab 10% and tocilizumab 50%); 5) SpA on anti-TNF monotherapy, 76%; 6) SpA on anti-TNF + MTX, 47%; and 7) SpA on NSAIDs/analgesics, 59%. The entire study population, single or two vaccine doses, showed a similar pattern, with SpA patients on anti-TNF as monotherapy having a significantly larger responder proportion compared to the other treatment groups except for SpA on NSAIDs/analgesics (Chi2/Fisher’s exact test; *P* between <0.001 and 0.033).

RA patients treated with rituximab (n = 10) showed a significantly impaired antibody response compared to all other treatment groups with only one patient showing a positive immune response.

Abatacept treated patients (n = 5) as a group showed a decreased antibody response compared to the other treatment groups. The limited number of patients precluded further analysis.

Only two RA patients treated with tocilizumab participated in the study. However, both patients were able to gain high postvaccination antibody titers comparable with those of SpA patients on anti-TNF as monotherapy or SpA patients receiving NSAIDs/analgesics.

### Immune response in relation to age

**Figure 4:**
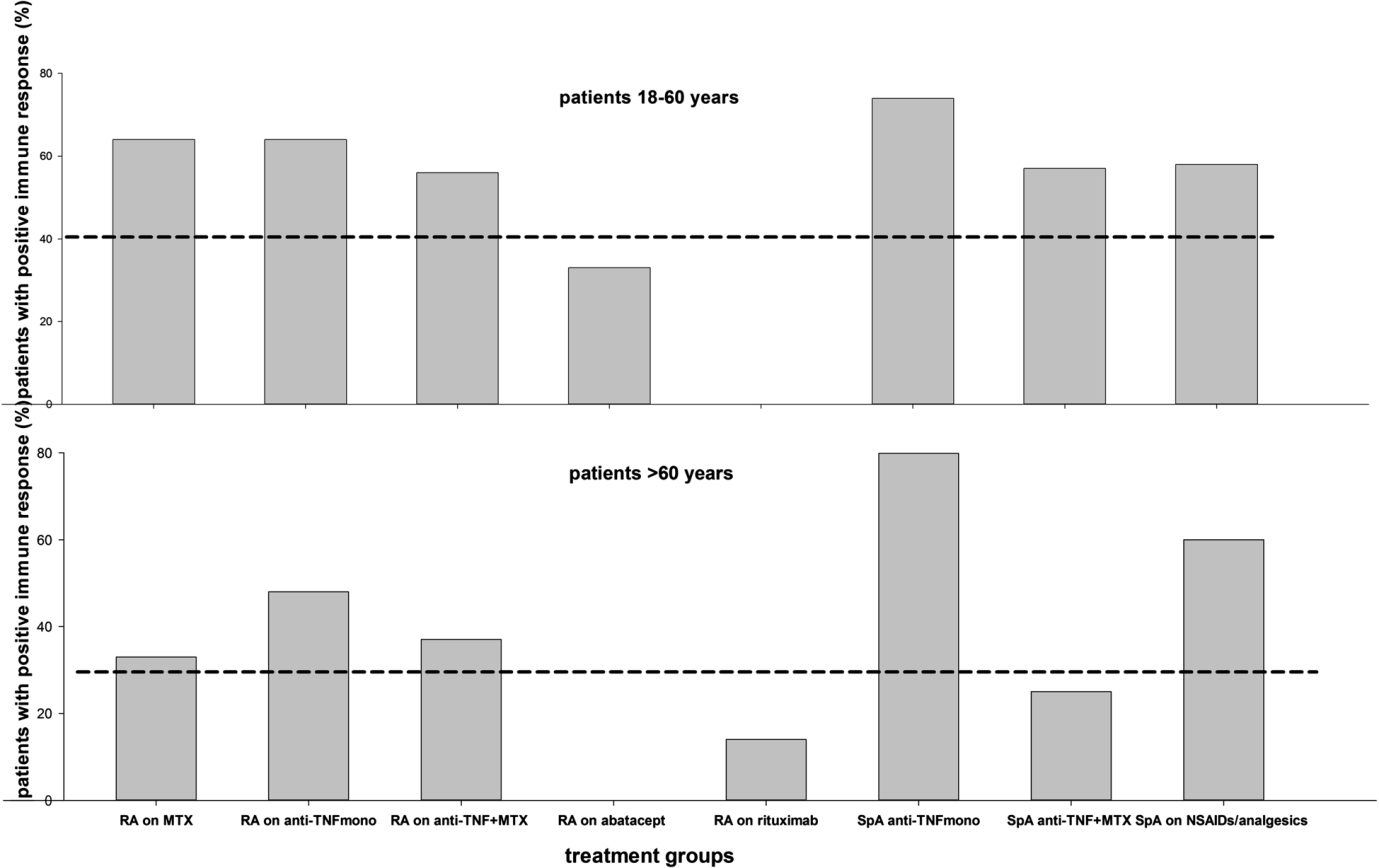
**The percentage of patients with a positive immune response, that is seroconversion, (defined as prevaccination titers <10 and postvaccination HI titers ≥40 or a ≥4-fold increase in HI titers) following vaccination against H1N1 influenza stratified according to age (18 to 60 years and ≥ 60 years).** The dashed lines denote seroconversion levels in healthy individuals when sufficient protection against infection is assumed (EU Committee of Human Medicinal Production criteria; CHMP). HI, hemagglutination inhibition.

Table 3 and Figure 4 summarize immune response in the entire study population and different treatment groups stratified for age, that is, subjects 18- to 60-years old (n = 142) and those ≥60-years old (n = 149). Regardless of age, rituximab treated patients as a group did not fulfill any CHMP serologic criteria for response. Furthermore, no abatacept treated patients <60 years met any of CHMP criteria. All other treatment groups fulfilled at least one serological criterion indicating protection against pH1N1 infection.

**Table 3:** Immune response following vaccination stratified according to age irrespective of number of doses

	**All patients (number = 291)**	**RA on MTX (number = 50)**	**RA on anti-TNF monotherapy (number = 38)**	**RA on anti-TNF + MTX (number = 53)**	**RA on abatacept (number = 5)**	**RA on rituximab (number = 10)**	**RA on tocilizumab (number = 2)**	**SpA on anti-TNF monotherapy (number = 41)**	**SpA on anti-TNF + MTX (number = 51)**	**SpA on NSAIDs/ analgesics controls (umber = 41)**
≥**60 years**										
**Patients (number)**	**149**	**36**	**27**	**35**	**2**	**7**	**1**	**10**	**16**	**15**
GMT prevaccination mean (95% CI)	8.1 (7.2 to 9.2)	8.6 (6.5 to 11.3)	9.5 (6.5 to 13.8)	6.3 (5.3 to 7.6)	----	6.1 (3.8 to 9.9)	---	8.7 (5.2 to 14.5)	12 (8 to 17.6)	7.2 (5.3 to 10)
GMT postvaccination mean (95% CI)	30 (24.3 to 37)	26.2 (17.2 to 39.9)	42.1 (22.7 to 78.1)	24.9 (16.8 to 36.8)	----	12.2 (5.5-27.2)	----	92 (46 to 184)	19 (11.7 to 31.4)	42 (18.6 to 95)
Geometric mean fold increase (95% CI)	3.7 (3 to 4.6)	3.1 (2 to 4.8)	4.4 (2.5 to 7.8)	3.9 (2.5 to 6.2)	----	2 (1.1-3.8)	----	10.6 (6.2 to 18)	1.6 (0 to 9-3)	5.8 (2.4 to 14.1)
Patients with prevaccination titer ≥40 (n;%)	9(6%)	5 (14%)	2 (7%)	1 (3%)	0	0	0	0	1 (6%)	0%
Patients with postvaccination titer ≥40 (seroprotection)	72(48%)	15 (42%)	15 (56%)	14 (40%)	0	2 (29%)	1 (100%)	8 (80%)	7 (44%)	10 (67%)
Patients with positive immune response (seroconvers.)	60( 40%)	12 (33%)	13 (48%)	13 (37%)	0	1 (14%)	1 (100%)	8 (80%)	4 (25%)	9 (60%)
% immunized with seasonal influenza vaccine (2009/2010)	51%	64%	56%	43%	50%	43%	100%	50%	56%	27%
**18-60 years**										
**Patients (number)**	**142**	**14**	**11**	**18**	**3**	**3**	**1**	**31**	**35**	**26**
GMT prevaccination mean (95% CI)	10 (8.7 to 11.4)	8.6 (5.5 to 13.5)	10 (6 to 16.7)	7.9 (5.7 to 11.1)	40 (7.1 to 224)	12.6 (1.7 to 92)	---	9.4 (7.1 to 12.4)	9.8 (7.5 to 13)	10.8 (7.5 to 16)
GMT postvaccination mean (95% CI)	58 (46.1 to 73)	98 (40 to 235)	66 (19 to 227)	39 (17.7 to 84)	64 (4.6 to 881)	8 (1.1 to 58)	---	72 (47 to 110)	51 (33 to 78)	60 (35.7 to 100)
Geometric mean fold increase (95% CI)	5.8 (4.5 to 7.6)	11.3 (4.4 to 29)	6.6 (1.9 to 23.5)	4.8 (2 to 11.6)	1.6 (0.2 to 12)	0.6 (0.1 to 4.6)	---	7.7 (4.5 to 13)	5.2 (3 to 8.8)	5.5 (3.2 to 9.5)
Patients with prevaccination titer ≥40 (n;%)	16 (11%)	1 (7%)	1 (9%)	0	2(67%)	0	1 (100%)	3 (10%)	3( 9%)	5 (19%)
Patients with postvaccination titer ≥40 (seroprotection)	95 (67%)	10 (71%)	7 (64%)	10 (56%)	2 (67%)	0	1 (100%)	23 (74%)	25 (71%)	17 (65%)
Patients with positive immune response (seroconvers.)	86 (61%)	9 (64%)	7 (64%)	10 (56%)	1 (33%)	0	1 (100%)	23 (74%)	20 (57%)	15 (58%)
% immunized with seasonal influenza vaccine (2009/2010)	20%	14%	9%	39%	33%	0	100%	13%	14%	27%

Positive immune response, that is seroconversion, was defined as prevaccination titers <10 and postvaccination HI titers ≥40 or a ≥4-fold increase in HI titers. CI, confidence interval; GMT, geometricial mean antibody titer; HI, hemagglutination inhibition; MTX, methotrexate; NSAIDs, non-steroidal anti-inflammatory drugs; RA, rheumatoid arthritis; SpA, spondylarthropathy.

### Predictors of positive immune response –univariate regression analysis (all patients)

Higher age was associated with an impaired positive antibody response (*P* < 0.001). Compared to RA more patients with SpA showed a positive immune response (*P* = 0.004). Ongoing MTX and rituximab were associated with an impaired immune response (*P* = 0.004 and *P* = 0.033, respectively). Prevaccination antibody titers were inversely associated with a positive immune response (*P* = 0.011). Smokers had a significantly lower antibody response (*P* = 0.020). Neither time period between vaccination and retrieval of blood samples nor immunization against seasonal influenza 2009/2010 influenced immune response to pH1N1 vaccine significantly.

### Multivariate logistic regression analysis (all patients)

The results of multivatiate logistic regression analysis including the time period between vaccination and collecting of blood samples are shown in Table 4. Higher age, higher prevaccination antibody titers and current smoking remained significant predictors of impaired immune response. The time between vaccination and retrieval of blood samples (months) did not have a significant impact on antibody response. The difference in response between patients with RA and SpA was not significant after adjustment in the regression model (Table 4).

**Table 4:** Predictors of positive immune response (seroconversion)*

	***P*-value**	**OR**	**95% CI**
**Age (years)**	<0.001	0.96	0.94-0.98
Sex (male/female)	0.984	0.99	0.57-1.73
Diagnosis (RA/SpA)	0.361	0.77	0.43-1.36
Time between vaccination and retrieving of blood samples (months)	0.334	0.97	0.91-1.03
**Current smoking (yes/no)**	0.025	0.47	0.24-0.91
**Pre-vaccination antibody titers**	0.001	0.97	0.94-0.99

Analysis was performed using a multivariate logistic regression model. CI, confidence interval; HI, hemagglutination inhibition; OR, odds ratio.

* Seroconversion is defined as prevaccination titres <10 and postvaccination HI ≥40 or a ≥4-fold increase in HI titers.

### Patients with negative prevaccination antibody levels

All patients with prevaccination antibody levels <10 were considered to have negative prevaccination serum, that is, not previously exposed to pH1N1 antigen (neoantigen). The percentage of patients with negative prevaccination serum in different treatment groups were: 1) RA on MTX, 19%; 2) RA on anti-TNF monotherapy, 16%; 3) RA on anti-TNF + MTX, 24%; 4) RA on other biologics (abatacept 1%, rituximab 4% and tocilizumab 0%); 5) SpA on anti-TNF monotherapy, 13%; 6) SpA on anti-TNF + MTX, 14%; and 7) SpA on NSAIDs/analgesics, 13%. Higher age, RA diagnosis and current smoking were associated with impaired antibody response (*P* < 0.001, *P* < 0.001 and *P* = 0.007, respectively) (univariate regression analysis). After adjustment in the multivariate logistic regression model, higher age remained a negative predictor of positive antibody response (*P* < 0.001) and current smoking showed a trend to a negative impact (*P* = 0.06). Corresponding to the results for all patients, differences in positive antibody response between SpA and RA were not significant. The time between vaccination and retrieval of blood samples (months) had no significant impact on antibody response in patients with negative prevaccination antibody levels.

### Safety of the vaccine

The vaccine was well tolerated and caused mostly mild to moderate side effects. Of 334 patients, 53 reported local pain and tenderness around the injection site, 41 patients had fever for a few days, and ten had influenza-like symptoms with muscle pain, headache and fatigue. A few patients experienced dizziness and upper airways infection. One subject developed pneumonia requiring treatment with antibiotics but not hospitalization.

Of 291 patients, 24 (8.2%) reported that vaccination influenced their rheumatic disease. The majority of these patients described more pain in their joints without objectively confirmed synovitis and six others reported increased morning stiffness and fatigue.

### Vaccination against seasonal influenza during the winter seasons 2009/2010 and 2010/2011

In total, 113 patients (34%) reported being immunized with seasonal influenza vaccine during the same winter season (2009/2010). Immunization with seasonal influenza vaccine (2009/2010) did not influence antibody response after pH1N1 vaccine significantly (univariate analysis). Forty three patients reported being vaccinated against seasonal influenza 2010/2011 at the sampling time and were, therefore, excluded from the main analysis. Of these 43 patients, 33 (77%) were women and 26 (61%) had RA. Mean time (range) between vaccination against pH1N1 influenza and sampling was 11(9 to 15) months. In total, 36 (84%) had protective antibody titers after vaccination and 34 (79%) had a positive antibody response (seroconversion).

### Patients who refrained from vaccination

In total, 70 patients refrained from the Pandemrix® vaccination. Of these, 46 (66%) were women and 43 (60%) had RA. Mean age and mean disease duration (range) in this group were 59.2 (25 to 87) and 17.3 (1 to 57) years. The distribution of these patients in different treatment groups (1 to 6) were: 1. 11%, 2. 20%, 3. 17%, 4. 3%, 9%, 1%; 4. 17% 5. 11% and 6.10%. Only five of these patients (7%) were vaccinated against seasonal influenza 2009/2010.

Blood samples were available in 26 of these patients. In total, three patients (11%) increased in antibody titers and reached protective levels in spite of not being vaccinated against pH1N1 influenza in sera collected after the vaccination campaign. Demographic characteristics did not differ significantly between patients who received vaccine against pH1N1 influenza and those refraining from vaccination.

## Discussion

We report on the influence of modern anti-rheumatic treatment on long-term immune response following vaccination with inactivated, monovalent, adjuvanted vaccine against pH1N1 influenza virus performed during the winter season 2009/2010 in patients with established arthritis. More than eight months after vaccination, the positive immune response in SpA patients on anti-TNF as monotherapy was still as good as that reported for healthy adults three to four weeks after vaccination [3]. Arthritis patients on MTX or anti-TNF combined with MTX had a lower immune response compared to responses reported for healthy adults but still met CHMP serologic criteria for protection against infection. Another important finding is that the antibody levels remained protective for a substantial time after pH1N1 vaccination without a clear diminishing pattern within the current follow up time frame.

Ongoing CD20 depleting treatment in RA using rituximab is associated with severely diminished immune response regardless of the patient’s age.

Interestingly, in RA patients on MTX treatment we did not observe an increased percentage of responders in those who received two vaccine doses compared to those who received one dose. MTX-treated patients younger than 60 years showed a better antibody response, with 64% of these patients having a positive immune response compared to 33% of these patients ≥60 years. This difference probably reflects the age-associated decline in immunity affecting both T- and B-cells (immunosenescence) [26,27]. Although immune responses in MTX-treated patients were lower than those reported for healthy adults, patients on MTX as a group meet at least one criterion required for protection according to CHMP [2-6,25]. Adler *et al*. found MTX to be a significant predictor of diminished immune response following pH1N1 influenza vaccine in patients with different inflammatory rheumatic diseases and none of the MTX-treated patients fulfilled CHMP criteria six months after vaccination. Apart from differences in the study population, patients participating in that study were treated with higher MTX doses given exclusively subcutaneously which might explain the diverging results [10]. In another study, MTX along with other disease-modifying anti-rheumatic drugs (DMARDs) was identified as a predictor of diminished response after immunization with Pandemrix® vaccine [13]. Elkayam *et al*. reported lower antibody response after a single dose of another adjuvanted H1N1 vaccine in patients with different rheumatic diseases compared to healthy controls but MTX was not associated with impaired immune response [14].

RA patients receiving anti-TNF treatment in the present study showed a lower antibody response compared to that of healthy adults (two to five) after a single dose of the vaccine, but boosting with an additional dose improved antibody response which is in accordance with results from the study of Gabay *et al*. [13]. Interestingly, antibody response was not significantly more impaired in RA patients treated with anti-TNF in combination with MTX compared to anti-TNF as monotherapy. More than nine months after immunization, RA patients on anti-TNF as a group regardless of age still met at least one criterion for protection according to CHMP. Also, anti-TNF treatment was not identified as a significant predictor of impaired antibody response in regression analysis.

The proportion of patients with positive immune response (seroconversion) among SpA patients on anti-TNF as monotherapy was as good as that reported in healthy controls after two doses of vaccine, both in patients <60 years old and those older than 60 years [2-5]. A second dose of the vaccine had a significant boosting effect in these patients. When anti-TNF treatment was given in combination with MTX the antibody response was significantly lower. The differences between the effect of anti-TNF on immune response in RA and SpA patients are in line with previously reported data [10]. Patients with SpA were on average younger than RA patients which could at least partly explain these differences. Since RA patients in general tended to have a lower immune response, the impact of the immunological disturbance as a part of RA disease could not be ruled out.

Our findings are quite different from those reported by Franco *et al*. in which SpA patients treated with infliximab or adalimumab had a diminished antibody response whereas RA patients on anti-TNF treatment had as good an immune response as healthy controls [11]. Patients participating in that study were immunized with a single dose of non-adjuvanted pH1N1 vaccine containing 15 μg HA. The usage of adjuvant and a booster dose of vaccine could explain the enhanced immune response among patients on anti-TNF treatment in our study. Our results are more in line with a Japanese study in which RA patients on anti-TNF treatment tended to have a lower antibody response compared to patients not receiving anti-TNF treatment [12].

A recent report in children with juvenile idiopathic arthritis (JIA) vaccinated against pandemic influenza showed that the antibody response overall was lower in patients with JIA, but neither MTX nor anti-TNF remedies affected the immune response significantly [15]. Among children with different rheumatic diseases only treatment with glucocorticoids was identified as a predictor of diminished antibody response [16].

Rituximab-treated patients immunized after several treatment courses showed somewhat lower pre-existing antibody levels compared to the other treatment groups. However, the ability of rituximab-treated patients to induce an antibody response was significantly hampered compared to other treatment groups reflected by lower postvaccination geometric mean titers (GML), seroprotection rate and seroconversion rate. These results are in line with previously reported data following vaccination against both seasonal and pH1N1 influenza [13,17,18]. B-cells require presentation of a protein antigen (included in the pH1N1 vaccine) to naïve T-cells for differentiation into antigen specific immunoglobulin (Ig) producing plasma cells [28]. Rituximab causes a shortage of mature B-cells with secondary diminished differentiated plasma cells and this may explain the decreased antibody response.

The number of abatacept-treated patients in the present study was limited but the seroconversion rate among this group was lower compared to other treatment groups with the exception of RA patients on rituximab. Abatacept-treated patients had significantly higher prevaccination antibody levels and seroconversion may underestimate whether there were true responses. However, a recently published study reported severely reduced immune response to pandemic influenza vaccination in 11 RA patients treated with abatacept [19]. Further studies investigating the impact of rituximab and abatacept on the immunogenicity of neoantigens are needed.

Only two tocilizumab treated patients participated in the present study. Both were immunized with two doses of the vaccine and responded with a satisfactory immune response well in line with a recent report of satisfactory antibody response in RA patients on tocilizumab [20].

Antibody response following vaccination diminishes during aging (immunosenescence). This is explained by changes in the immune system affecting both B- and T-cells [26]. A rapid decline of protective antibody levels four months after seasonal influenza vaccination has also been reported in the elderly [27]. Patients younger than 60 years who participated in the present study had significantly better antibody responses compared to those older than 60 years. Higher age was identified as a predictor of impaired antibody response in the univariate analysis and also remained after adjustment for diagnosis, disease duration, smoking status and prevaccination antibody titers in the multivariate regression analysis. Current smoking was associated with diminished antibody response. We found smoking to be associated with an impaired antibody response following pneumococcal vaccination in the same patient cohort [29]. Our results are in line with a recent study in which smoking was negatively associated with persistence of seroprotection 12 months after immunizaion against pH1N1 influenza using the AS03 adjuvanted vaccine in HIV-infected adults [30].

Patients immunized against seasonal influenza during 2010/2011 showed higher seroprotection and seroconversion rates compared to other patients. This indicates that seasonal influenza vaccine containing pH1N1 virus strain was able to boost immune response in these immunosuppressed arthritis patients.

During the 2009 influenza pandemic in Sweden vaccination was performed using vaccine containing the AS03 system [2]. This adjuvant has been shown to enhance the antibody response to inactivated pH1N1 vaccine in both younger and older adults [5]. The impact of the adjuvant on the immunogenicity of the vaccine may at least partly explain the diverging results in the present study compared to those reported after vaccination with unadjuvanted influenza vaccine [3,11,19,30].

Strengths of the present study are that the analyses were blinded for demographic and treatment data and the standardized blood sampling as part of a vaccination study in arthritis patients treated in clinical practice. Limitations include possible recall bias of vaccination status (retrospectively collected information) and the fact that antibody response is a surrogate marker of protection. The numbers of patients treated with biological remedies other than TNF-antagonists and those in different groups after categorization for diagnosis, treatment or doses of pH1N1 vaccine was too limited to allow multiple comparisons between groups. Furthermore, the considerable proportion of patients with pre-existing antibody levels limits the ability to detect a positive response. However, analysis including patients with negative prevaccination levels (that is, titers <10) for whom pH1N1 represents a neoantigen did not show diverging results compared to the entire study population. Our data would suggest that approximately 10% (3/26) of non-vaccinated patients with negative prevaccination levels who reached protective levels of pH1N1 were exposed to pH1N1 antigen during the current observation period.

The variable time periods of postvaccination samples collection limits comparison with other studies assessing antibody response four to six weeks after vaccination. On the other hand, we demonstrated persistence of protective antibody titers several months after immunization.

In spite of these weaknesses, our results reflect the cross-sectional picture of arthritis patients met in daily rheumatologic clinical practice.

Immunosuppressed patients with arthritis are recommended yearly influenza vaccination but antibody response following vaccination is not routinely measured. Results from the present study confirm that the majority of these patients (except those on rituximab) would reach sufficient serological immunity and are expected to be protected against the infection. For clinicians taking care of these high risk patients, our results may be used as a support to recommend influenza vaccination.

## Conclusions

Overall, our data support that vaccination yields serological indications of longstanding protection against pH1N1 infection in a large proportion of arthritis patients. Protective antibody titers could be detected for up to 22 months after vaccination in the current patient population, with the exception of rituximab- (and possibly abatacept-) treated patients.

## Abbreviations

anti-TNF: Anti-tumor necrosis factor inhibitors; CHMP: Committee of human medicinal products; GMT: Geometric mean antibody titers; HA: Hemagglution inhibition assay; HI: Hemagglutination inhibition; JIA: Juvenile idiopathic arthritis; MTX: Methotrexate; NSAIDs: Non-steroidal anti-inflammatory drugs; pH1N1: Pandemic H1N1 influenza; RA: Rheumatoid arthritis; SpA: Spondylarthropathy.

## Competing interests

The authors declare that they have no competing interests.

## Authors’ contributions

MCK participated in the design of the study, performed the statistical analyses and wrote the manuscript. TS, LEK, AM and PG conceived of the study, participated in its design and coordination and helped to draft the manuscript. TA performed the analyses. All authors read and approved the final manuscript.
